# Childhood disabilities and the cost of developmental therapies: the serviceprovider perspective

**DOI:** 10.1080/17482631.2024.2345816

**Published:** 2024-04-24

**Authors:** Sabika Shaban, Hira Amin

**Affiliations:** a College of Islamic Studies, Hamad Bin Khalifa University, Doha, Qatar; b College of Public Policy, Hamad Bin Khalifa University, Doha, Qatar

**Keywords:** Children with disabilities, neurodevelopmental disabilities, disability costs, health care providers, disability in Qatar

## Abstract

**Purpose:**

For children with neurodevelopmental disabilities (CWNDs), early diagnosis that leads to early intervention with regular targeted therapies is critical. In Qatar, private therapy centres that address this demand often have highly exclusive prices restricting families from availing them. This paper examines the challenges faced by families with CWNDs, as well as various financial and systemic obstacles, from the vantage point of these centres, all of which culminate in an extraordinarily high disability price tag for disability families in Qatar.

**Methods:**

This study is based on qualitative, semi-structured, and in-depth interviews with private therapy centres and developmental paediatricians.

**Results:**

Therapy centre representatives expressed common struggles in lengthy and cumbersome administration and licencing procedures, difficulty in hiring and retaining high quality staff, and expenses that need to be paid to the state. From their experience, families largely struggle with delayed diagnoses that significantly slow down intervention plans and therapies as well as staggeringly high financial costs with a dearth of funding options.

**Conclusions:**

We recommend sincere engagement, dialogue, and cooperation between multiple stakeholders; a supportive ecosystem to balance and distribute the demand that includes schools and parents; as well more efficient administrative procedures and recruitment strategies.

## Introduction

Managing disabilities is a complex endeavour. Yet there are factors that can make a significant difference to a person with disabilities’ (PWD) life outcomes, opportunities, and quality of life for themselves and their caregivers. Particularly for children with neurodevelopmental disabilities (henceforth CWNDs), early diagnosis and regular targeted therapies are pivotal during the period of optimum brain development in the initial years of their life (Sharpe & Baker, 2007; Vietze & Lax, 2018). Studies show that intervention at the earliest possible moment produce better outcomes, including preventing regressions in developmental growth as well as inhibiting secondary developmental challenges (Alamdarloo & Mradi, 2020; Eliso, 2017; Mengoni & Oates, 2014). However, the initiation and the frequency of therapies for CWNDs are highly dependent on the availability of the service, but more so on the cost factor, which is decidedly expensive. This is especially true given that therapy interventions are an intensive service requiring sophisticated processes and methods in a predominantly one-to-one interaction setup consistently over a period of years.

In Qatar, the number of private centres that provide therapies, such as speech or cognitive therapy and psychological counselling, often have highly exclusive price lists that restrict many families from availing them (Shaban & Amin, 2023).^1^ This typically results in detrimental impact on the CWND’s developmental progress and wellbeing. Through qualitative, semi-structured, and in-depth interviews, this paper explores the disability price tag for families with CWNDs^2^ in Qatar from the point of view of private therapy centres. Although CWNDs are the subject of this study, many of the findings apply to CWDs in general. The “disability price tag” is the extra cost that parents with a CWND would have to pay to ensure the same standard of living as a neurotypical child. This paper is part of a larger study on the disability price tag in Qatar from various stakeholder perspectives that are currently in review or in press (e.g., Shaban & Amin, 2023). This particular paper focuses on private therapy centres. It is important to look into the financial dimension from their perspective to gain a better understanding on why there is such a perversion between the supply and demand of developmental interventions in the country.

The paper first provides a brief overview of disability laws, policies, and services in Qatar. This is followed by the methodology and then the results which examine the therapy centres’ challenges and constraints as well as their experiences with families with CWNDs. The paper concludes by discussing possible strategies and ways forward.

## Developmental support in Qatar

The State of Qatar has been engaged in an ongoing and ambitious strategic exercise in aligning its Qatar National Vision (QNV) 2030 with the United Nations’ Agenda 2030 to jointly tackle mutual strategic areas of interest. Persons with disabilities (PWDs) comprise an important element of Sustainable Development Goal (SDG) 10 on Reduced Inequalities and overall has strong interlinkages with all other SDGs. Qatar ratified the UN Convention on the Rights of the Child (UNCRC) in 1995 and was also one of the first countries to ratify the UN Convention on the Rights of Persons with Disabilities (CRPD) in 2008 (PSA, 2021). Qatar’s commitment has also been reflected in various public events and initiatives,^3^ the most prominent being hosting the most accessible World Cup for fans with disability in FIFA history (InsideFIFA, 2022). Qatar will also host the Global Disability Summit in 2028 in cooperation with International Disability Alliance (QNA, 2022).

One of the obligations of the UN’s CRPD (2006, p. 19 Article 26) on its signatory states is to provide tailored developmental services early on. It says that states must:

… organize, strengthen, and extend comprehensive habilitation and rehabilitation services and programmes, particularly in the areas of health, employment, education and social services, in such a way that these services and programmes … begin at the earliest possible stage, and are based on the multidisciplinary assessment of individual needs and strengths.

Exhibiting similar goals, the Qatar Voluntary National Review 2021 highlights the intention of the Ministry of Public Health (MOPH) to incorporate the rights of PWDs “in all aspects of the national health strategy” (Peninsula, 2021). In fact, one of the priorities of the National Health Strategy is “The Health and Wellbeing of People with Special Needs,” in which it commits to ensuring sufficient care services for PWDs, improving the level of support provided to families of PWDs, and improving accessibility within schools for CWDs (MOPH, 2018, p. 19).

Despite these successes, there are numerous issues—the key concern being that the number of PWDs and CWDs in Qatar have been significantly underreported. The census bureau records about 2,400 children as having a disability in Qatar (i.e., under the age of 19; UN ESCWA, 2018). However, the same census records the number of PWDs under the age of 19 years that access private therapy centres total as nearly 3,000. This figure does not account for those CWDs who are financially unable to access services in the first place. Neighbouring states in the Gulf that share similar socioeconomic features proclaim significantly higher disability prevalence rates. These all indicate that the census falls short by a very large margin to be able to effectively inform policymakers of the scale of the disability community residing within Qatar’s borders. The report entitled *Child Well-Being in Qatar* also highlights this point stating that disability figures do not align with the evidence of a much higher prevalence rate of childhood disability—most especially given the high incidence of consanguineous marriage in the country (PSA, 2010). Other reasons for underreporting are a lack of trained developmental diagnosticians, low awareness, and cultural hesitancies in seeking professional support (DIFI, 2018).

Inaccurate figures and flawed methodologies to gather data are deeply problematic as public resources are allocated, measured, and tracked to determine progress based on these figures and methods. For example, the National Strategy’s national target by 2022 is statistical: “By 2022, 20% improved access to the health care services for persons with disabilities and special needs” (MOPH, 2018, p. 35) Another poignant example is the Qatar Rehabilitation Institute (QRI). In an effort to increase capacity, the Qatar Voluntary National Review 2021 mentions QRI as one of the products of the National Health Strategy: “It is the largest centre in the Arab region for persons with disabilities that provides various services, including community and career rehabilitation programs and research” (Peninsula, 2021). However, it seems that the centre has been designed based on insufficient statistical data as it falls far short of the actual scale of need. The most telling indicator of this, anecdotally through qualitative queries conducted with families raising CWDs in a related study (Shaban & Amin, 2023), are the long waiting lists as well as an average of two-week gaps between each therapy session, which significantly reduces the effectiveness for the vast majority of PWDs and CWDs according to the developmental paediatricians and therapy centre representatives. Moreover, the number of private therapy centres have increased from 9 centres in 2009 to 35 centres^4^ in 2019 to help absorb some of the unmet needs of CWDs (Brik, 2021). Therefore, without accurate data and identification of pitfalls, work towards a better public system is markedly difficult.

Another pertinent issue is the lack of clarity around services and rights for non-citizen residents. Qatar is a unique country where expatriates significantly outnumber local citizens (Cochrane et al., 2024). Managing PWDs and CWDs amongst short- and long-term expatriate workers who contribute to the development of the country, yet do not pay government tax, needs to be clearly addressed and managed with dignity, according to the international treaties and policies Qatar has ratified and pledged to uphold. Currently, when legislation or reports explicitly state PWDs or CWDs they are referring to Qatari citizens, and it remains unclear the extent of state support towards its larger non-Qatari resident population-one example being the QNV 2030 tracking reports. In the last report entitled *Measuring the Standard of Living in Qatar* (2015), data refers mostly to the improved conditions and future aims targeting the Qatari minority, with rare mentions of how the state addresses the standards of living for non-Qatari residents. Laws are also generic with no explicit mentions of non-Qatari residents (“Law No. 2 of 2004 in Respect of People with Special Needs,” 2004). The latest National Health Strategy does mention residents but restricts its plans to “long-term residents who require assistance for disabilities that may be medical, mental, or psychological.” Yet, it does not specify what “long term” means or how the needs of “short-term residents” who have disabilities will be addressed.

That being said, the situation is not entirely unique to Qatar. Financial accessibility to developmental services as well as other social access issues tend to be a typical and very serious barrier for PWDs around the world (Amin et al., 2024; Badran et al., 2023a, 2023b, 2023c; UNDESA, 2018). In Qatar, however, cost is compounded with inadequate insurance coverage and low capacity which greatly exacerbates the situation (Sharpe & Baker, 2007). Past studies have also observed inaccessibility to developmental care provision due to physical, attitudinal, informational, and communication issues (UNDESA, 2018). Kheir et al. (2012)’s study on Qatar’s ecosystem confirm the same concerns, such as “long waiting lists, inconsistent provision of services, inadequacy in respect to staff numbers and expertise, complicated processes of referral, and sometimes language barriers between the service providers and service beneficiaries.”

This study updates the current state of developmental support offerings from the perspective of private therapy centres that provide developmental therapy services to CWDs. It explores their multiple challenges in depth and possible pathways forward.

## Methodology

The Institutional Review Board (IRB) of the Qatar Biomedical Research Institute (QBRI) ethically reviewed and approved this research (QBRI-IRB 2021-11-111). The study was conducted using multiple methods. The initial stage started with quantitative surveys modelled on the Goods and Services Required Approach (Mont & Cote, 2020) with fifteen diverse families with CWNDs followed by semi-structured interviews with the same families as well as some of the main stakeholders in Qatar: ministry officials, private school leaders, developmental paediatricians, and private therapy centres. This holistic, multi-stakeholder approach was to ensure different perspectives were captured. In terms of data analysis, the data was transcribed and coded according to stakeholder roles and inductively analysed to identify themes. This was done using the six phase Braun and Clarke (2012) method: familiarizing yourself with the data; generating initial codes; searching for themes; review potential themes; define and name themes; producing the report. Author one took the lead in this process in discussion with author two. This paper only discusses the methodological details and key findings from the last two categories, namely developmental paediatricians and private therapy centres, although it is broadly informed by the entire dataset. If required, a strong quote from the other stakeholders may be included to emphasize a particular point. The last two categories included seven founding owners and senior managers of therapy centres, essentially representing 20% of centres in Qatar, and two of a very limited number of developmental paediatricians in the country that serves CWDs—their profiles are outlined in Table I. The bulk of the research in this paper focuses on private therapy centres’ experiences with a few details that were checked and confirmed with the developmental paediatricians. The remaining data from the other sources are currently in the process for publication.

**Table I.** Profile of the interviewees.

Therapy Center Representatives Profile	n=7
Licensing Body	
MOEHE	4
MOPH	3
Financial Tier*	
High	1
Medium	2
Low	4
Interviewees	
Owners	2
Managers	5
Developmental Pediatricians	*n*=2
**Total Interviewees**	**9**

*Tiers for the private therapy centres are categorized simplistically by gauging the price of the daytime programs and independent therapy sessions.

Author one, who has been a disability activist for the last ten years in Qatar and is thus familiar with the local context and field, conducted these nine interviews from late 2021 to early 2022. In qualitative research, there is no set sample size, but data saturation is typically a satisfactory indicator of an ideal number. With regards to the focus area for the study, data saturation was reached when “data adequacy” was achieved (Morse, 1995)—that is, common themes emerged between the interviews but by the ninth interview no further significant themes were identified with regards to experiences of family encounters, the lack of support from the state, and the kind of opportunities through which the disability ecosystem can be enriched. While anecdotes continued to be diverse in every interview, the themes that connected them remained fairly similar within each interview—which was unsurprising given that they encompassed a sizable proportion of therapy centres within a small state. Interviews comprised three areas of discussion: (1) the basic setup of the centre, such as licencing body, price lists of their services, and regulation from ministries; (2) their engagement with client families, including discussions or negotiations around fees charged to families; and (3) their views on the current financial ecosystem for both families and private therapy centres, which included their understanding of the gaps that they feel remain unaddressed and the opportunities available to make the ecosystem more financially sustainable.

All participants signed a consent form in advance and were aware that they could opt out at any time. Data is either presented in aggregate form or with very few identifier markers to maintain anonymity. Interviews were between 30 to 60 minutes. Although COVID-19 restrictions had been removed at the time, eight interviews were conducted via the Zoom platform or the phone depending on the preference of the interviewee. One interviewee preferred to attend the interview in person.

## Results

The results have been divided into two overarching themes. First are challenges faced directly by private therapy centres. This can be further divided into three sub-themes: (a) dealing with the state, which includes incognizant state administration, duplicate licencing pathways, and expenses to be paid to the state; (b) challenges in the recruitment and retention of human resources; and (c) delayed diagnoses that directly impact intervention plans. The second overarching theme are the challenges and the limitations of the options faced by the families with CWNDs who use these services, yet from the vantage point and experiences of the private centres. This also can be further sub-divided into three sub-themes: (a) high financial costs, (b) lack of other financial options, and (c) reliance on alternative methods such as parental training. The first-hand experiences of the families of private therapy centres have been documented in other publications by the authors, but the researchers found that they strongly corroborate with the centres’ perspectives below.

## Challenges faced by therapy centers

### State administration, licensing pathways, and expenses

Centres providing intervention support for CWNDs incur high financial expenses, which translate into high price schedules for their clients. According to one manager, “many centers are struggling to survive … . Many centers are in debt.” Another centre administrator admits that its owners heavily contribute “from personal finances every month” to not compromise on their quality of services.

The support from the state remains limited or negligible in the context of a disability-catering industry in the private sector: “we are treated just like another commercial entity—there is no relief.” Licencing departments demand extensive requirements for renewal of the centre’s licence, and each step incurs an administrative cost. “There is no need [for the state to] give money [to us] but at least do not take money from us—QAR 1,000 here, QAR 3,000 there … we are here serving special needs!” expressed one representative. By the time the process is complete, within 6 months the centre restarts the full process for the next year. One interviewee expressed the frustration to at least extend the cycle to two or three years to allow them some stability and free up resources to concentrate on developing their clients.

Centres providing therapies have two licencing pathways, each of which has different requirements but ultimately provide similar services. In this study, four centres have been licenced by the Ministry of Education and Higher Education (MOEHE) while three are licenced by MOPH. When asked what is the difference between both licencing systems, the responses varied. “In terms of the services [provided by the center], it is the same,” one manager said. “They’re just different paths, different standards, different requirements.” One of the founding owners mentioned that MOEHE-licenced centres have a heavier emphasis on academic content and operates like a school. However, the description of the daytime programs or early intervention programmes were fairly similar across all the centres. One manager mentioned that there are more stringent measures when licenced by MOPH, where the practitioners and their qualifications, the venues and their health and safety protocols, and the programmes on offer are all rigorously scrutinized. At the same time, a MOEHE-licenced centre mentioned the considerable involvement of the ministry in approving their curricula and staff—even as far as interfering in routine operational matters. In both cases, the rigour of licencing seemed fairly high and interventionistic. One centre representative also mentioned a recent possible update that all practitioners within MOEHE-licenced centres may need to get authorization to practice by MOPH, potentially intensifying the administrative demand. As an additional factor, certain types of professionals such as speech pathologists have far lower licencing requirements in order to practice in Qatar compared to international standards^5^—which has an unfortunate impact on the quality of services that are delivered locally.

In all cases, price lists are sent to the licencing ministry for approval as part of the annual process of licence renewal. Four interviewees confirmed that the ministries have never rejected any changes in the prices; it is unclear what criteria is used by the responsible department or if changes in pricing over the years are tracked before approving price lists, opening up questions about how the prices charged to their clients are regulated. Overall from the interviews, it remains obscure as to why there is a duplicate licencing pathway for therapy centres, where outwardly they offer nearly identical services to the disability community. It also remains unconfirmed whether the licencing departments within each ministry coordinate their efforts to have a comprehensive understanding of the wider disability service industry.

The role of individuals within state departments was also emphasized. One centre representative remarked: “Not every [one of the many processes and procedures] is documented and not everything is published. It all depends on the man sitting on the counter at that particular time and the rules are made by him depending on how well his day has been.” Another comment mentioned was as follows: “We have wonderful leadership coming up with great ideas and concepts, and we have great platforms, and we have a wonderful QNV 2030…. In spite of all that, the people on the ground, the ones on the front lines, they are not sensitized to all of this. They take the role of God and you are my dependent. That needs to change. It’s an attitudinal change.”

In addition, a centre representative commented on the allocation of state money, which was echoed by two other interviewees:

I attended an autism event. It had cost I think QAR 10 million to put together. With that, I was thinking, I could reduce my session price to be 30 percent of the current price. If you generalize what is being spent on some events and redirected to an actual impactful program, it would be much better.

Although Qatar is one of the richest countries in the world and spends generously on events, this quote illustrates how some therapy centre representatives felt that this was an inefficient usage of funds. This money, they argue could be better utilized elsewhere such as reducing their costs that would in turn help them reduce their prices. Three out of seven centres specifically mentioned rent as a critical expense, which has placed spatial constraints on their capacity to accommodate more CWNDs. Two centres indicated that there are schemes amongst the ministries in Qatar that offer rent relief to corporate entities if they align with specific strategic priorities of the state, but such rent-relief schemes for therapy centres that help advance the disability ecosystem in the country has unfortunately not materialized.

### Human resources: recruitment and retention

All seven centres fervently expressed their frustrations with human resources (HR) concerns—both in general in terms of finding and retaining suitable candidates as well as the lack of support in the recruitment process by the state. These have had direct impact on their financial status and their ability to support their clients fully within the amount they charge. In terms of finding expert practitioners, “it takes me a year to find a suitable candidate,” “I cannot find therapists locally—it never happens,” and “we pay a lot for recruitment agencies, and even they struggle to find suitable candidates who are willing to come to the [Gulf Corporation Council] GCC.” Centres which aim to keep their fees as low as possible struggle even more with inflexible salary offers in order to maintain the price schedule. While this may be an industry-wide issue globally, what seems to aggravate the situation is the role of the state in HR that was felt by a majority of the interviewees: “I am not free to choose applicants from different nationalities. I am governed by a list of nationalities given to me by [state departments].” Centres choosing to change the mix of nationalities in order to reflect the cultural mix or language needs of their clientele face significant issues in trying to hire from outside the nationality list.

In addition, quality is an instrumental factor in the healthcare industry and most especially when dealing with children. It is also an unfortunate correlation that, according to the interviewees from private centres, high quality comes with high salary packages to attract and retain practitioners. This is partially related to more stringent licencing requirements that ensure higher quality of training, which tend to exist in countries where labour cost is also correspondingly higher than in the Middle East, as alluded to earlier in the paper. Furthermore, the global demand for practitioners is high and local capacity is severely lacking,^6^ requiring recruiters to further increase salary packages in order to provide enough of a pull factor for international candidates to relocate to the GCC. As a result, payroll and HR-related expenses remain one of the largest—if not the largest—expense category for all the centres that were interviewed.

### Diagnostics

Typically, therapy centres receive CWNDs who have either been diagnosed—which leads to more concrete intervention plans; or without diagnoses, at which point assessments are recommended based upon which intervention plans are designed. The key catalyst to a strong intervention programme is an official diagnosis. The public healthcare provider, Hamad Medical Corporation (HMC), and Sidra Medicine^7^ are looked at as the leading diagnostic centres in the country. However, the responses of the seven centre owners and managers towards this have been as follows:

These wait lists? I had a child referred to Rumailah [HMC] at age 2 years and 1 month, and I called the mother and she said she got the diagnosis after one year. We lost all that time in waiting and potential early intervention time.

They don’t realize the importance [of early diagnoses] and how scary it is. When parents come with red flags, you must start intervention immediately.

“I had a child who is extremely aggressive and can induce harm. They need medication in order to manage him but it cannot be given without a diagnosis. They told her last week [January 2022] to come in December”. [2022]

Some of the interviewees felt that MOPH needed to rethink some of their systems and reduce the centralization of the diagnostic process. One strategy proposed was to cut down waiting lists by training junior medical professionals in a basic red flags system and not require a formal diagnosis in order to enter the CWND into HMC’s developmental programmes. Another interviewee pointed out that these developmental programmes only last for a few months before exiting the CWND from the system, and even then, sessions are only provided every other week: “Surely it is not enough for our children.” So, the centre representative continues, “why are we talking about waiting lists [for diagnoses] with 22 centers for special needs in Qatar? Why not make a group of managers of these centers and have a deal to support [the demand for diagnoses and therapies]?” Ultimately, there is much to be done in order to increase capacity of provisions for the many CWNDs who continue to lack access to adequate interventions.

## Challenges faced by families with CWNDs

### High financial costs

All centres offer a daytime programme for groups of CWNDs that incorporates therapeutic interventions, socialization skills, and academic content over a period of 3 to 5 hours. The fee structure charged to families for the daytime programme can range from QAR 3,000 to QAR 20,000 per month. This range is fairly broad, dictated by multiple factors as stated by our interviewees, including frequency of individual therapies in a given week within the programme, the qualifications and background of specialists, the target clientele (families from low, medium, or high income brackets), and operational costs (such as rent, equipment, and administrative fees).

The centres also provide a menu of services after hours or on Saturdays, including an assortment of individualized therapies such as speech therapy, occupational therapy, physical therapy, behavioural therapy, applied behaviour analysis (ABA) therapy, and psychological counselling. Between the seven centres interviewed, these range from 40 minutes to an hour per session, at the rate of QAR 250 to QAR 400 per session.

Therapeutic interventions are meant for extended periods of time. The ideal “formula” for different levels of need is challenging to develop as the requirements are so diverse. As an approximate guide, however, the developmental paediatricians that were interviewed suggested three to seven assorted therapy sessions a week for a CWND with moderate needs, with the caveat that these are arbitrary estimations that need continuous reassessment as the child develops. However, given even a wide-ranging estimate with a wide-ranging price schedule for interventions, a CWND with *moderate needs* can incur anywhere between QAR 3,000 to QAR 11,200 per month on therapies alone.

Interviewees were asked if or how often finance came up as a topic of discussion between the centres and their clientele, and the nature of these discussions. One centre mentioned that there was a diversity of family situations in their centre: those with government support, coverage from employers, limited coverage from insurance, different income brackets, and so on.

However, most of the responses from both centre management as well as clinicians referred to scenarios that troubled them the most:

We provide some free consultations to parents [and then they tell us]: “We wish we could bring our children here but we cannot take the cost.”

Sometimes our costs are more than the salary of the father—it is impossible for them.

It is very common to hear [about financial struggles] … . Even from the Qataris … not all the time do they get allowances or financial support.

We always talk about the financial struggle, because now the salaries in Qatar are not like before.

It is a very, very sad situation. I have a few mothers who have more than one child with special needs. And the husbands are working at very entry-level jobs … .

Fifteen to twenty percent of prospective parents [that come to us]—can even be more—cannot afford to even start [therapies].

But I know sometimes the residents, but even the locals, take their children out because it is too much.

The quotes demonstrate the everyday struggle of centre management to respond to these situations, where they find themselves delicately navigating the opposing forces of a business that aspires to serve the community but faced with very real obstacles in making their services accessible to economically vulnerable families. Two of these interviewees stated their efforts to engage with ministry officials, but with little progress.

In general, “the special needs sector in Qatar is expensive—especially for expats.” The centre representative explains: “The school fees is insane. Especially special education. A minimum they charge is QAR 90,000 per year—this is the minimum…. In addition to the schools, they need to pay the shadow teacher (educational assistant who works with the child/children in an educational setting providing personalized support). In addition to that, they need to pay for therapies … . Most of the parents pay from their pockets.” Overall, all centre representatives rued the situation that many CWNDs were forced to experience, most especially due to financial barriers.

### Lack of financial options

As one centre manager states: “You know parents don’t come here for 1 month or 2 months; they come here for years.” Consequently, six centres stated that they “try to keep the fees as low as possible; but there is a limit.” These limitations involve meeting the operational expenses of the centres and providing salaries that are attractive enough to secure experienced talent. Each of the seven centres shared anecdotes about how they have tried to support families generally, as well as support specific cases where families were simply unable to pay the fees.

One centre provides discounts based on memberships, for example membership with Qatar Autism Society. Another mentions: “The maximum Finance [Department] allows is 10% [discount]. If I do more, I will start losing money.” One interviewee shared how during the pandemic, in response to massive job losses or salary cuts of their clientele, they dropped the fees for their individualized therapies by 30%, charging just enough to break even. Some centres have designed packages and advanced payment mechanisms that provide parents with discounted rates from 10 to 40%. One centre provides different options to families such as prioritizing one type of intervention or reducing number of sessions just enough to allow progress—albeit slow, but “at least [they] will not fully back out.” Yet another centre ensures providing winter clothes, diapers, and other basic necessities to client families in need when they simply cannot afford them. Two centres have mandated a significant percentage of their profits to be categorically utilized for underprivileged families. Ultimately, all of these initiatives, while well-intentioned, remain “band-aid” solutions to an inherently systemic concern of families losing out on therapies for their CWNDs.

When it comes to accessing financial aid, five centres mentioned providing their client families with referrals to charitable organizations, most especially Qatar Charity and Zakat Fund. One was unsure if Qatar Red Crescent provides any support. One mentioned Eid Charity as an excellent option, while another mentioned that it no longer provides the support. These referrals may be for families with very low incomes, high family expenses, have more than one CWD, or a combination of these factors. One interviewee stated that being a non-Muslim may impact the eligibility of the applicant—though this claim remains unverified. Two centres mentioned that cases referred to charities are typically entertained if the family income is equal to or less than QAR 10,000. However, if the income exceeds QAR 10,000—regardless of how many dependents this income supports or the severity of the financial demand—the likelihood of receiving support is less. As one centre representative explains, “the father has still all the same expenses and more: the rent, the school, the therapies, other children … so where does he go [for help]?” This speaks to the fact that agencies may need to consider the conversion handicap and be willing to evaluate full family contexts before utilizing a static income figure as a screening criterion for eligibility.

Two interviewees explicitly mention the extensive paperwork involved as the process is “very long and very complicated,” which can become overwhelming for parents to collate. In the course of the study, other centre managers not in this specific study made multiple remarks on other barriers, such as no formal instructions available regarding the process, instructions where available are mostly in Arabic, and lack of accessibility to officials by centres to ask questions. Three centre representatives mentioned the challenge of time—the length of time involved in applying for the fund, receiving the fund, and the duration of time of coverage, providing only temporary relief.

The application process involves the submission of financial documents and medical reports. The centre has to add supporting documents to the application such as an acceptance letter for the child’s registration and their own proof of licence. By two to four weeks after the complete application is submitted, an internal committee within the donor entity reviews the case and agrees to provide partial or full coverage for a set period of time (typically three to six months). One centre did mention that it is easier to renew the arrangement for another cycle once the parent is registered in the system.

Once the case has been accepted, issuing payments can also result in delays; for instance, one centre mentions: “I have one child who has been waiting for 5 months for the cheque from the charity.” Another mentions: “The wait list can be like a year before the money starts coming.” Conversely, one centre mentioned it can take minimum three weeks: “To be frank and honest, it depends on the families’ connections in these organizations.” In spite of the duration, two centres specifically praise the organizations for being “very, very supportive,” while one centre representative was more critical of the support.

Some of the responses by centre representatives included the following:

I know a few parents who went there and they provided … it is not a huge amount… they need a lot of paperwork and time… but it helps.

Sometimes I advise the parents: let us start and let’s wait for one to two months while the charity processes [their application]. Sometimes the parents get scared we will start demanding payment when the charity takes too long [to process].

You come across families that say “me and my spouse decided we need to get quality for our two children. We do not need anything for us. We do not need savings. We need to give them the quality they need.”

We have children from very poor families … . We have been losing a lot due to [financially] unable parents.

One centre mentioned registering with some insurance companies, another mentioned their select clients availing insurance coverage, but yet another interviewee mentioned that insurance companies do not provide any disability-specific coverage and in fact have specific clauses that exclude coverage for any expenses related to one’s disability. The last most corroborates with the collective knowledge of the fifteen parents; that is, no insurance company was found that would cover disability-related interventions. In fact, one of the centre representatives stated that there is a strong need for support in encouraging the insurance industry to provide disability-specific provisions.

Lastly, two centres mentioned utilizing personal networks, professional relationships, and corporate sponsorships to support some of their needs. These individual philanthropists and corporate entities have helped provide some financial support through short-term sponsorships of enrolled clients, and non-financial support—such as surplus wheelchair stocks, depreciated furniture, or IT equipment—to mitigate some of the operational expenses; however, these materialize on an ad hoc basis.

### Parental training: an alternative

Centres in Doha continue to battle “lack of knowledge” and “the Google search” amongst parents through readjusting expectations and countering misinformation. Three centres explicitly mentioned their strategy to empower parents with information and skillsets on how to manage their CWNDs. They noticed parents with training were “more engaged” and “very successful” in dealing with their CWNDs. The centres consider their parental training programmes as a slow but effective tool that can alleviate some of the need for interventions if parents are better equipped to carry on the training at home and understand the impact of their steps. This in turn can mitigate some of the detrimental impact on the CWND if financially strained parents need to reduce the number of therapy sessions.

In fact, one interviewee shares that some parents tend to remove their child from the centre at the first instance of showing improvement, so that they can save up on the cost of therapies by continuing the intervention care at home on their own. This usually slows down any further progress, but the centre tries its best to support however possible, whether through complimentary trainings or providing resources to use at home: “We are trying to do what we can.”

## Discussion and policy recommendations

Therapy centre representatives expressed common struggles in terms of lengthy and cumbersome administration and licencing procedures, difficulty in hiring and retaining high quality staff, and expenses that need to be paid to the state. From their experience, families largely struggle with delayed diagnoses that significantly slow down intervention plans and therapies as well as staggeringly high financial costs with a dearth of funding options. There was an almost unanimous agreement that there can be very simple and practical ways that the state can contribute to the viability of the disability service industry.

A comprehensive approach to disability would involve engaging multiple stakeholders. Three interviewees mentioned the importance of more collaboration, cooperation, and communication channels between the state and private sector players: “This country has amazing people … everyone has good intentions but going it alone. [We all] need to sit on one table.” Cooperation and discussion could lead to industry-wide standards for clinicians, qualifications, and the quality of services, to enable some degree of regulation and standardization between centres with different licences. Another interviewee mentioned that discussions with multiple stakeholders had existed at one point in late 2000s, when the conversation around developing a national autism strategy had been dynamic and interesting programmes were being designed. One such programme was an “autism fund” that would allow families raising children on the spectrum to supplement developmental care with the private sector without additional charge to parents. Many of these discussions have failed to materialize, but the discussion frameworks are still available and can be revived for further development and implementation. Ultimately, one centre representative argues that the sincerest way forward would be for the state to really “listen to us and … dig deep into real issues and then do something about it … [while] giving us protection against voicing our challenges—after all, it is the same ministries that we need to go back to [for our routine operations].”

Furthermore, one centre stated the need to think more creatively by designing and supporting programmes that support therapy centres serving this sector. A wider supportive ecosystem can help re-balance the load and alleviate some of the pressure points. Financial relief organizations and initiatives for both the centres and the families with CWDs are perhaps the most important. These can include sustainable and long-term criteria-based funding, financial relief schemes, and favourable loan terms for large-scale expenses. Ministries can design programmes to provide financial support like rent relief through a “social enterprise” framework to ease the current transfer of cost to their clientele (EC, 2015; ILO, 2017). A new Qatar healthcare insurance law (Law No. 22 of 2021) is currently being implemented in stages; however it is not clear if this insurance would cover specialist medical care, therapies, and equipment that CWDs require regularly (ITA, 2022).

Another key part of the wider system is schools (Amin & Cochrane, 2023; Amin et al., 2023; Romanowski et al., 2023). For instance, one centre owner states: “If I was a decision maker, international school licenses would not come without accepting 25 [CWNDs] within your building. If every school was forced to do so, we would not even need [this center].” Enforcing all schools to have inclusive education and offer therapies would ease some of the pressures from the developmental centres as well as reduce the strain on parents from continuously taking their CWDs to multiple places and pay multiple fees. It is recommended that the MOEHE and MOPH work closely together to design bridges between developmental therapy centres and mainstream schools in both governmental and private sectors to facilitate more CWNDs entering private education. They can jointly design programmes that engage therapy centres in the private sector to meet the gaps both in public sector provision for healthcare but also with mainstream schools in the private sector.

Parents with CWNDs are also a crucial part of this ecosystem who must be acknowledged, informed, and engaged. Therefore, it is recommended that the MOPH adopt more child- and family-centred procedures in which families receiving new diagnoses are provided comprehensive information and resources by medical teams that are trained in exhibiting empathy and cultural sensitivity (DIFI, 2018). State departments in general require more awareness and training into the needs of CWNDs: “Still in the government they do not understand or believe what is this therapy.” According to one interviewee, the MOPH can also lead in establishing comprehensive parental training programmes beyond just autism to mitigate mental health concerns of families raising CWNDs as well as empowering them with appropriate tools and management skills (DIFI, 2018); the ministry can utilize knowledge resources from the private sector to support such programmes.

Finally, administrative procedures should be streamlined. It is recommended that ministerial departments redesign some of their licencing processes to elongate the renewal cycle, allow greater flexibility in visa applications, enable legitimacy of online degrees for recruitment—especially in light of new educational trends sparked by the pandemic, and reduce waiting times between different administrative processes. There can further be more concentrated effort in providing educational degree programmes to produce locally trained practitioners in both Arabic and English to reduce the current strain of recruiting qualified professionals from outside the country.

## Conclusion

This paper explored the multiple financial and systemic obstacles private therapy centres face in Qatar that usually result in charging high prices and at times offer inadequate or insufficient services. Several challenges were identified: cumbersome or inconsistent state administration; duplicate licencing pathways; expenses to be paid to the state; difficulty in recruitment and retention of human resources; and delayed diagnoses that directly impact tailored and effective intervention plans. From their vantage point, families with CWNDs often have difficulties with the high financial costs, lack of financial options, and a lack of alternative methods such as parental training being offered. This leads to parents delaying or having less frequent therapy appointments for their children, at best, or not having therapy altogether at worst, leading to negative outcomes for the CWND. Based on the research, we have three recommendations. First, there needs to be sincere engagement and dialogue with the multiple stakeholders between state officials and practitioners. Second, attention needs to be given to cultivate a supportive ecosystem to redistribute the financial and service demand, such as insurance or funds dedicated for schools and inclusive education. Parents also need to be fully informed and integrated within this ecosystem. Third, administration, application of processes and procedures, and licencing pathways need to be streamlined; recruitment and retention of high-quality staff should be made easier; as well as effort should be dedicated to building formal capacity and professional training for the resident population in both English and Arabic.

## Notes

1. The cost to the family is highly dependent on the type and extent of disability as well as individual family dynamics. Please see (Shaban & Amin, 2023) for more detail on precise figures as well what these costs mean as a percentage of income for different families.

2. Children with neurodevelopmental disabilities may or may not have physical disabilities. Hence, children with only physical disabilities (e.g., blind, deaf, or mobility-aid-using children who have neurotypical capacities) are excluded from this study.

3. These include: the most recent Population Policy of the State of Qatar 2017–2022 issued in 2015 which identifies empowering persons with disabilities as one of its pillars; Qatar’s National Autism Plan (2017–2021); and the Cabinet’s decision in 2019 to establish a National Committee for Women, Children, Elderly, and Persons with Disabilities Affairs. Yet, there remains many roadblocks such as CWDs are not recognized as a separate category, there is little clarity on the actualization of implementation plans, and non-Arab citizens of Qatar are not included in discussions.

4. It is worth studying the availability of interventions for adults with disabilities, as almost all of the therapy centres mentioned here cater to PWDs under 18 years of age. Anecdotally, adults, most especially with neurodevelopmental disabilities, seem to have very limited options available.

5. As illustration, a BA graduate in speech pathology from Qatar University can be licenced upon graduation and having completed an internship within the program; whereas in the UK, licencing is issued upon a Master’s degree in addition to two years of supervision. See as example: Department of Healthcare Professionals (DHP), Ministry of Public Health (MOPH), *Circular No. (24/2020)*, 28 Dec 2020, https://dhp.moph.gov.qa/en/QCHPCirculars/Circular%20(24–2020)%20-%20Eng.pdf; and Ministry of Public Health (MOPH), *Psychology Scopes*, n.d., https://dhp.moph.gov.qa/en/Documents/Psychology%20Scopes.pdf; and “Speech and Language Therapists”, *Health and Care Professions Council (HCPC)*, https://www.hcpc-uk.org/standards/standards-of-proficiency/speech-and-language-therapists/.

6. At present, there are scarce opportunities for education and training of high-quality practitioners across all fields in Qatar, with almost negligible opportunities available in English. This is further exacerbated by the lack of recognition of online degrees, which is often the only form of educational training open to residents in Qatar who want to contribute to the field.

7. Essentially, HMC is the only official diagnostic centre; however, diagnoses made within Sidra Medicine are accepted in many contexts once it is authenticated by HMC.
